# A Closed-Loop Modeling Framework for Cardiac-to-Coronary Coupling

**DOI:** 10.3389/fphys.2022.830925

**Published:** 2022-02-28

**Authors:** Anneloes G. Munneke, Joost Lumens, Theo Arts, Tammo Delhaas

**Affiliations:** Department of Biomedical Engineering, CARIM School for Cardiovascular Diseases, Maastricht University, Maastricht, Netherlands

**Keywords:** coronary circulation, coronary hemodynamics, transmural myocardial flow, computational model, cardiac-to-coronary coupling

## Abstract

The mechanisms by which cardiac mechanics effect coronary perfusion (cardiac-to-coronary coupling) remain incompletely understood. Several coronary models have been proposed to deepen our understanding of coronary hemodynamics, but possibilities for in-depth studies on cardiac-to-coronary coupling are limited as mechanical properties like myocardial stress and strain are most often neglected. To overcome this limitation, a mathematical model of coronary mechanics and hemodynamics was implemented in the previously published multi-scale CircAdapt model of the closed-loop cardiovascular system. The coronary model consisted of a relatively simple one-dimensional network of the major conduit arteries and veins as well as a lumped parameter model with three transmural layers for the microcirculation. Intramyocardial pressure was assumed to arise from transmission of ventricular cavity pressure into the myocardial wall as well as myocardial stiffness, based on global pump mechanics and local myofiber mechanics. Model-predicted waveforms of global epicardial flow velocity, as well as of intramyocardial flow and diameter were qualitatively and quantitatively compared with reported data. Versatility of the model was demonstrated in a case study of aortic valve stenosis. The reference simulation correctly described the phasic pattern of coronary flow velocity, arterial flow impediment, and intramyocardial differences in coronary flow and diameter. Predicted retrograde flow during early systole in aortic valve stenosis was in agreement with measurements obtained in patients. In conclusion, we presented a powerful multi-scale modeling framework that enables realistic simulation of coronary mechanics and hemodynamics. This modeling framework can be used as a research platform for in-depth studies of cardiac-to-coronary coupling, enabling study of the effect of abnormal myocardial tissue properties on coronary hemodynamics.

## Introduction

With the ever increasing load of coronary heart disease, a better understanding of factors that influence coronary function is of utmost clinical importance. Although blood flow and pressure in the proximal epicardial arteries have been studied extensively (Ofili et al., 1993, 1995; Akasaka et al., 1996; Crowley and Shapiro, 1997; Daimon et al., 2005; Davies et al., 2006; Hadjiloizou et al., 2006, 2008; Okura et al., 2006; Tani et al., 2009), microcirculatory data is scarce as clinical measurements, particularly non-invasive ones, are difficult to perform and limited in resolution. Mathematical models, together with experimental studies, have played an important role in deepening our understanding of coronary hemodynamics, providing useful insights into, e.g., the effect of myocardial contraction and intramyocardial pressure on coronary flow waveform (cardiac-to-coronary coupling), pressure wave propagation in epicardial coronary arteries, large microvascular compliance, and transmural differences of flow patterns (Arts and Reneman, 1985; Burattini et al., 1985; Beyar and Sideman, 1987; Bruinsma et al., 1988; Flynn et al., 1992; Ge et al., 2018).

Reported models of the coronary circulation differ largely in form and degree of complexity. The majority of the early models have been of the lumped-parameter [zero-dimensional (0D)] type, testing the key mechanisms by which coronary flow is determined (Suga et al., 1973; Downey and Kirk, 1975; Spaan et al., 1981; Arts and Reneman, 1985; Burattini et al., 1985; Beyar and Sideman, 1987; Krams et al., 1989). The major limitation of the 0D modeling approach is that wave propagation effects, particularly those arising from cardiac contraction (Guiot et al., 1990), are neglected. More recently, therefore, several one-dimensional (1D) models have been proposed to study coronary perfusion by prescribing a time-varying external pressure on subendocardial vessels (Guiot et al., 1990; Rammos et al., 1998; Mynard et al., 2010, 2014; Algranati et al., 2011) or alternatively by coupling the coronary circulation to a left ventricular (LV) time-varying elastance model in an open-loop (Reymond et al., 2009; Duanmu et al., 2019; Scarsoglio et al., 2019) or closed-loop manner (Fan et al., 2020, 2021). Mynard and Smolich (2015, 2016) as well as Ge et al. (2018, 2019) described both left and right ventricular mechanics with a time-varying elastance model coupled to a detailed anatomically based coronary network. Although the above-mentioned 1D models are perfectly suitable to study the effect of valvular and vascular pathologies on coronary flow hemodynamics, a detailed study on cardiac-to-coronary coupling is limited as mechanical properties like myocardial stress and strain are not taken into account. Hence, this approach makes it difficult to study the effect of cardiac pathologies like mechanical dyssynchrony on coronary flow hemodynamics.

The CircAdapt model of the human heart and circulation originally focused on cardiac mechanics and hemodynamics, relating global ventricular pump mechanics to local myofiber mechanics with the one-fiber model for myocardial contraction (Arts et al., 2005). After integration of modules describing mechanical ventricular interaction (Lumens et al., 2009), (heterogeneity in) mechanical behavior in myocardial tissue segments (Walmsley et al., 2015), and 1D wave propagation (Heusinkveld et al., 2019), the multi-scale CircAdapt model enables real-time beat-to-beat simulation of cardiovascular mechanics and hemodynamics in a wide range of physiological and pathophysiological situations. Examples are heart failure with preserved ejection fraction (van Loon et al., 2020), myocardial scar (Huntjens et al., 2014), and mechanical dyssynchrony (Walmsley et al., 2015). A modeling framework that couples a coronary model to the CircAdapt model would enable modifications of myocardial properties per wall (segment), allowing a different cardiac-to-coronary coupling for each coronary vascular bed simulated. Hence, such a modeling framework would be perfectly suited to study the effects of the above-mentioned cardiac pathologies on coronary perfusion.

Therefore, we implemented a model of coronary mechanics into the well-established CircAdapt model to construct a modeling framework that couples ventricular cavity pressure and local myofiber mechanics to the intramyocardial pressure and coronary flow, enabling future in-depth study of cardiac-to-coronary coupling. The anatomical configuration of the coronary circulation was chosen such that the model complexity remains minimal, while still capturing relevant details at each level of the network; the large coronary vessels were modeled one-dimensionally, whereas small vessels were represented by lumped elements. Flexibility of the coronary model was shown by simulation of the influence of aortic valve stenosis on coronary flow.

## Materials and Methods

### Overview of the Model

**FIGURE 1:**
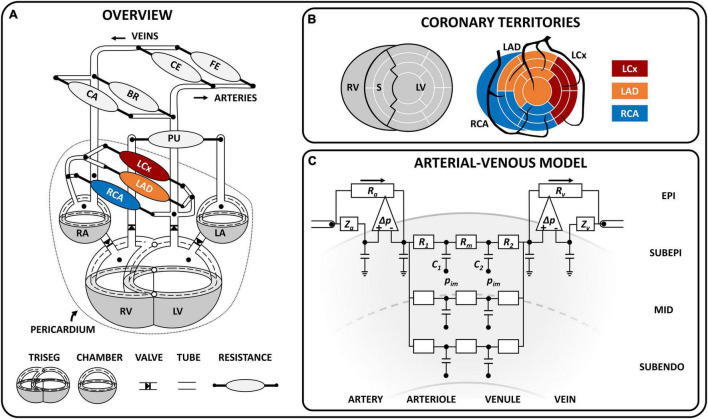
Schematic model overview. **(A)** Schematic representation of the closed-loop cardiovascular CircAdapt model, including the TriSeg module (Lumens et al., 2009) [which allows mechanical interaction at the junction (°) between the left (LV) and right (RV) ventricle and the septal (S) wall], the chamber module (Arts et al., 2005), valve module (Palau-Caballero et al., 2016), one-dimensional tube module (Heusinkveld et al., 2019), and zero-dimensional arterio-venous resistances for systemic and pulmonary circulation (Arts et al., 2005) as well as coronary circulation. The dashed line indicates the midwall volume; the dotted line indicates the pericardium, which surrounds the heart and coronary circulation. The coronary circulation included three major branches, namely the left circumflex (LCx), left anterior descending (LAD) and right coronary (RCA). **(B)** Schematic overview of the territory assignment of the three main coronary branches. The LV and S wall were divided into 12 and 5 segments, respectively, using the MultiPatch module (Walmsley et al., 2015). Coronary territories of the LCx, LAD and RCA were assigned to individual segments according to the AHA proposed 17-segment model (Cerqueira et al., 2002). **(C)** Coronary arterial-venous model with epicardial arterial inlet and venous outlet accounting for wave behavior in the connecting 1D vessel. The pressure difference-controlled flow source (Δ) accommodates high flow conditions (i.e., low peripheral resistance) and makes solving the differential equation for this highly non-linear system less complicated. The arterial-venous model is subdivided into three transmural layers (subepicardium, mid, subendocardium), each containing an arteriolar and venular compartment (with compliances *C_1_* and *C_2_*, resistances *R_1_* and *R_2_*, and a shared resistance *R_m_*). The intramyocardial pressure (*p_im_*) varies between layers and acts on the compliance. BR, brachial; CA, cerebral; CE, celiac; FE, femoral; LA, left atrium; RA, right atrium. Symbols: *R_a_*, arterial resistance; *R_v_*, venous resistance, *Z_a_*, arterial wave impedance, *Z_v_*, venous wave impedance.

The coronary module was integrated in the closed-loop CircAdapt (Arts et al., 2005; Lumens et al., 2009; Walmsley et al., 2015; Heusinkveld et al., 2019) computational model of the cardiovascular system to simulate coronary hemodynamics.^1^ The CircAdapt model allows fast beat-to-beat simulation of cardiovascular mechanics and hemodynamics in both healthy and (patho-)physiological conditions (Huntjens et al., 2014; Walmsley et al., 2015; van Loon et al., 2020). The multi-scale CircAdapt model consists of a network of different modules representing the main elements of the closed-loop cardiovascular system, including cardiac walls, local cardiac tissue mechanics, cardiac valves, pericardium, large blood vessels, and systemic and pulmonary circulation (Figure 1A). In the next few sections, we will describe the most relevant aspects of the cardiac module and we will introduce the governing equations, modeling assumptions and implementation of the new coronary module in detail. A description of the remainder modules can be found in Arts et al. (2005), Lumens et al. (2009), Walmsley et al. (2015); Heusinkveld et al. (2019).

### Cardiac Module

The cardiac module consists of five walls, i.e., the left and right atrial walls, the left and right ventricular walls and the interventricular septum (Figure 1A). The one-fiber model of myofiber mechanics was previously described (Arts et al., 1991; Walmsley et al., 2015) and relevant equations for this study can be found in Supplementary Material. Briefly, local myofiber mechanics are related to global pump mechanics under the assumption that myofiber tension is homogeneously distributed throughout the cardiac walls (Arts et al., 1991) as follows. Cavity volume stretches the myocardial wall, determining wall area and hence, myofiber strain. A model of myofiber mechanics is used to calculate myofiber stress from myofiber strain. In each cardiac wall, tension is determined by total myofiber stress, which equals the sum of active stress as a result of sarcomere contraction and passive stress originating from microstructural tissue components. With the TriSeg module (Lumens et al., 2009), mechanical ventricular interaction was established by force equilibrium in the junction of the three ventricular walls. Transmural pressure is calculated from wall tension and wall curvature using Laplace’s Law. Cavity pressures are calculated by adding the transmural pressures to the intrapericardial pressure induced by the non-linearly compliant pericardium surrounding the myocardial walls. Model-predicted cavity pressures have been extensively validated previously (Lumens et al., 2009; Walmsley et al., 2015).

With the MultiPatch module (Walmsley et al., 2015), the LV free wall and septum were subdivided into 12 and 5 wall segments, respectively, in order to assign individual myocardial segments to specific coronary territories (Figure 1B) according to the AHA proposed 17 segment-model (Cerqueira et al., 2002).

### New Coronary Module

The coronary circulation was modeled similar to the systemic circulation, in which large arteries and veins were represented by 1D tubes coupled to 0D arterio-venous models representing the microcirculation. The level of detail in the present coronary circulation model (e.g., number of 1D branches and 0D intramyocardial layers) was chosen with the objective of describing both epicardial and transmural myocardial sites of the coronary circulation in a simple manner, taking into account the available clinical and experimental data. The resulting coronary module was influenced by the work of Spaan et al. (2000) and Mynard et al. (2014).

#### One-Dimensional Modeling of the Coronary Vascular Tree

The coronary vascular tree was assumed to consist of four epicardial arteries and five epicardial veins (Figure 1A), modeled as 1D non-linear elastic tubes with a fixed length and variable cross-sectional area, to account for flow distribution and pulse wave propagation. The coronary arterial tree stems directly from the ascending aorta, consisting of (1) the left main (LM) coronary artery, which bifurcates into (2) the left circumflex (LCx) and (3) the left anterior descending (LAD) coronary artery, and (4) the right coronary artery (RCA). The distal ends of the left and right coronary veins were connected to opposite sides of the distal coronary sinus, which then drains by means of the proximal coronary sinus into the right atrium. The tube module with 1D wave propagation was previously described (Heusinkveld et al., 2019). Briefly, the wave behavior can be derived from the principles of conservation of mass and impulse momentum under the assumptions that blood flow in medium to large sized (coronary) vessels behaves incompressible and Newtonian (Ku, 1997). Parameter values for the 1D coronary tree can be found in Supplementary Material.

#### Lumped Parameter Modeling of the Intramyocardial Vessels

The terminal end of a coronary artery was coupled to a non-linear arterial-venous lumped model as shown in Figure 1C. The arterial-venous model has been described previously (Arts et al., 2012) and was extended to consist of three series-arranged compartments, namely an epicardial arterial compartment, intramyocardial compartment and epicardial venous compartment. Both the epicardial arterial and venous compartments consist of a pressure-dependent wave impedance, compliance and resistance. Separation of wave behavior and resistance in the large vessels facilitates modeling of the non-linear behavior considerably. Due to the transmural variation in intramyocardial pressure from epicardium to endocardium, each intramyocardial compartment was further divided in three myocardial layers according to penetrating depth (i.e., subepicardial-, mid-, and subendocardial layer). Each layer consists of an arteriolar and venular subcompartment with volumes depending on the compliances *C_1_* and *C_2_*, respectively. The arterial and venous subcompartments each contain a resistance (*R_1_* and *R_2_*, respectively); a middle resistance (*R_m_*) was shared by both subcompartments. The connection between the model of cardiac mechanics and the coronary circulation is made through the intramyocardial pressure (*p_IM_*) acting on the intramyocardial compliances (*C_1_* and *C_2_*) as shown in Figure 1C. Finally, the distal end of the arterial-venous lumped model was coupled to a coronary vein.

##### Myocardium-Vessel Interaction

**FIGURE 2:**
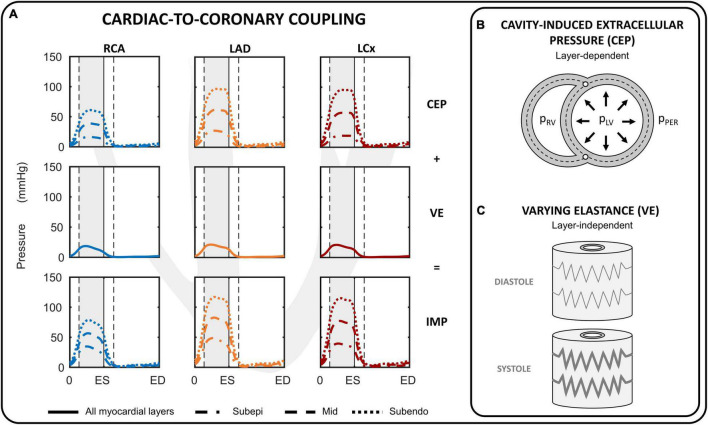
Cardiac-to-coronary coupling. **(A)** Overview of the intramyocardial pressure (IMP) during one cardiac cycle for the right coronary artery (RCA), left anterior descending (LAD) and left circumflex (LCx). The IMP is assumed to consist of two components: cavity-induced extracellular pressure (CEP) and myocardial stiffness (labeled as varying elastance; VE). **(B)** CEP arises from transmission of cavity pressure in the myocardial wall and varies linearly from cavity pressure (p_LV_, p_RV_) at the endocardial border to pericardial pressure (p_PER_) at epicardial border for the left and right ventricular free walls. **(C)** VE was directly related to fiber stress. ED, end-diastole; ES, end-systole.

The intramyocardial pressure is assumed to be time-varying and dependent on both cardiac contraction and transmural depth (Figure 2A). Increased intramyocardial pressure during systole is transmitted across the vessel wall, impeding perfusion particularly in the subendocardial layers. The intramyocardial pressure (*IMP*) was assumed to be a summation of two mechanism: 1) transmission of cavity pressure into the myocardial wall (cavity-induced extracellular pressure (*CEP*); Figure 2B), and 2) effect of myocardial stiffness (labeled as varying elastance (*VE*); Figure 2C). *CEP* was directly related to ventricular cavity pressure and assumed to vary linearly from ventricular cavity pressure (*P_1_*) at endocardium to pericardial pressure (*P_2_*) at epicardium. For the septal wall, *CEP* was varied linearly between LV pressure (*P_1_*) and right ventricular pressure (*P_2_*). *VE* was assumed to be related to fiber stress (σ_f_) as fiber stiffening increases myocardial pressure (Krams et al., 1989). Correspondingly, *IMP* was prescribed to be the sum of *CEP* and *VE* as follows:


(1) $$I\,M\,P=\underset{C\,E\,P}{\underbrace{\left(r\cdot P_{1}+\left(1-r\right)\cdot P_{2}\right)}}+\underset{V\,E}{\underbrace{\gamma \,\sigma_{f}}}$$

where γ is a prescribed scaling parameter, and *r* is the radial position of the subepicardial-, mid- and subendocardial layer prescribed at 1/6, 3/6, 5/6 of the myocardial wall segment, respectively. The *IMP* per coronary branch was calculated from a volume-weighted average of the *IMP* per coronary territorial myocardial wall segments. Parameter γ was chosen to produce a peak *VE* equal to ∼20% of peak LV pressure because in animal experiments subendocardial peak intramyocardial pressure generally exceeds peak LV pressure by 10–30% (Rabbany et al., 1989; Mihailescu and Abel, 1994). Note that the same value of γ = 0.06 was chosen for the left, right and septal walls.

##### Transmural Pressure

The transmural pressure of the intramyocardial vessels is equal to intravascular pressure minus intramyocardial pressure. The transmural pressure is related to vessel cross-sectional area by a non-linear power-law to phenomenologically capture the experimentally observed non-linear relation between transmural pressure (*p_trans_*) and area (*A*) in vessel walls as a result of its tissue components, collagen and elastin (Arts et al., 1991):


(2) $$p_{t\,r\,a\,n\,s}\left(t\right)=p_{0}\left({\left(\frac{A\,\left(t\right)+0.5\,A_{w}}{A_{0}+0.5\,A_{w}}\right)}^{k/3-1}-\max{\left(0,{\left(\frac{A\,\left(t\right)}{A_{0}}\right)}^{0.7\cdot \left(\frac{A_{w}}{A_{0}}\right)-0.4}-1\right)}^{2}\right)$$

At reference pressure *p_0_*, the cross-sectional area of the vessel lumen and vessel wall area are equal to *A_0_* and *A_w_*, respectively. The second term between brackets in Equation 2 was added to cope with negative transmural pressures and was based on the collapsible tube law (Shapiro, 1977; Kozlovsky et al., 2014). Note that with volume approaching the state of collapse, transmural pressure becomes progressively negative. The reference pressure (*p_0_*) was based on data reported by Spaan (1985).

##### Vascular Resistance

Based on the assumption that the resistance of each of the compartments depends on volume according to Poiseuille’s Law (Bruinsma et al., 1988), the volume-resistance relationship was


(3) $$R=R_{0}\,\frac{V_{0}^{2}}{V^{2}}$$

Where *R_0_* is the reference resistance and *V_0_* refers to the equal distribution of the proximal and distal reference volumes. Distribution of resistance over the epicardial arterial (*R_a_*), intramyocardial (*R_1_* + *R_m_* + *R_2_*), and epicardial venous (*R_v_*) compartment was assumed to be 28%:65%:7% during normal resting conditions (Chilian et al., 1989). For the intramyocardial resistances, the distribution over the arterioles, capillaries and venules (i.e., *R_1_* : *R_m_* : *R*_2_) was assumed to be 60%:30%:10% (Chilian et al., 1989).

The reference resistance (*R_0_*) was controlled by the target flow (*q_0_*) and reference pressure drop (△ *p_0_*) as follows


(4) $$R_{0}=\frac{△\,p_{0}}{q_{0}}$$

The reference arteriolar resistance (*R_0,1_*) was determined by adjusting the reference pressure drop (△ *p_0,1_*) to achieve target mean flow (*q_0,1_*), creating an “auto-regulated” healthy control simulation. The coronary target flow was distributed among myocardial segments in proportion to their myocardial weights (Table 1), with an subendocardial-to-subepicardial flow ratio of 1.11 (Crawford et al., 2009). Total weight of the left, septal and right walls were taken to be 114, 47, and 41 grams (myocardial density of 1.055 g/ml), respectively.

**TABLE 1:** Volume distribution based on myocardial weight and coronary territory.

	% RV wall	% S wall	% LV wall
LCx			42
LAD		60	33
RCA	100	40	25

### Simulation Protocol and Data Analysis

**FIGURE 3:**
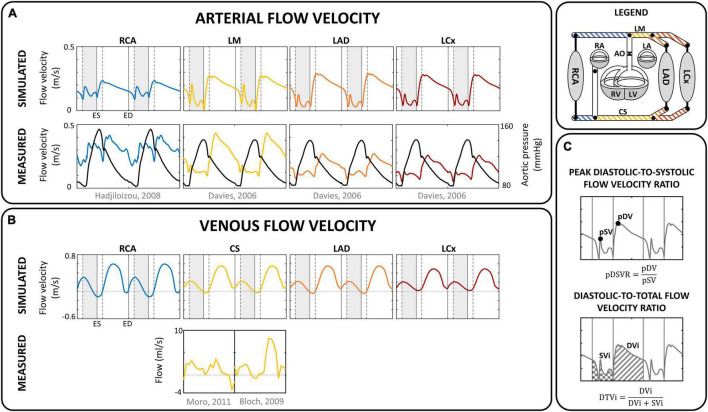
Flow velocity. Comparison of simulated **(A)** arterial and **(B)** venous flow velocity with measured flow (velocity) in corresponding sites for a single human subject (Davies et al., 2006; Hadjiloizou et al., 2008; Bloch et al., 2009; Moro et al., 2011). Vertical gray areas indicate left ventricular ejection and vertical dashed/solid lines indicate opening/closing of left ventricular cardiac valves. **(C)** Analysis of coronary flow waveform, in terms of peak diastolic-to-systolic flow velocity ratio (pDSVR) and diastolic-to-total flow velocity time integral (DTVi) as reported in Table 3. CS, distal coronary sinus; DVi, diastolic velocity integral; ED, end-diastole; ES, end-systole; LA, left atrium; LAD, left anterior descending coronary; LCx, left circumflex coronary; LM, left main coronary; LV, left ventricle; pDV, peak diastolic velocity; pSV, peak systolic velocity; RA, right atrium; RCA, right coronary artery; RV, right ventricle; S, septum; SVi, systolic velocity integral.

The reference simulation was modeled with the parameters listed in Table 2. Systemic parameters were chosen to yield representative function curves for a 70 kg adult at resting conditions. To validate the model framework, simulated coronary flow, velocity and diameter were compared to measurements obtained in human subjects. Specifically, arterial flow velocity waveforms of the LM, LCx, LAD, and RCA simulated by the model were qualitatively compared to flow velocity measurements performed by Davies et al. (2006) and Hadjiloizou et al. (2008). Quantitative comparison of arterial flow velocity with clinical data was conducted in terms of peak diastolic-to-systolic flow velocity ratio (Ofili et al., 1995; Akasaka et al., 1996; Crowley and Shapiro, 1997; Daimon et al., 2005; Okura et al., 2006; Hadjiloizou et al., 2008; Tani et al., 2009) and diastolic-to-total velocity time integral (Ofili et al., 1993, 1995; Crowley and Shapiro, 1997; Hadjiloizou et al., 2006, 2008) as shown in Figure 3C. Due to the scarcity of coronary venous data, validation of venous flow velocity was restricted to a qualitative comparison with measured coronary sinus flow waveforms (Bloch et al., 2009; Moro et al., 2011). Subendocardial flow and diameter were qualitatively compared to measured subendocardial LAD tracings (Hiramatsu et al., 1998; Kajiya and Goto, 1999), as intramyocardial RCA and LCx data are lacking. Furthermore, simulated transmural diameter, in terms of end-diastolic to end-systolic diameter, was quantitatively compared to LAD diameter changes measured transmurally (Kanatsuka et al., 1989; Nellis and Whitesell, 1989; Yada et al., 1993, 1995; Hiramatsu et al., 1994, 1998).

**TABLE 2:** Reference input parameter values.

	Unit	Reference value
**General**		
Heart rate	bpm	71
Cardiac output	L/min	5.1
Mean arterial pressure	mmHg	91
Systolic duration	%	41

**Coronary target flow**		
Right coronary artery	mL/min	112
Left anterior descending	mL/min	83
Left circumflex	mL/min	60

**Endo-to-epi flow ratio**		
Right coronary artery	–	1.11
Left anterior descending	–	1.11
Left circumflex	–	1.11

**Cardiac**		
Wall volume		
Right ventricular free wall	cm^3^	39
Septal wall	cm^3^	45
Left ventricular free wall	cm^3^	108

**Valve**		
Aortic valve orifice area	cm^2^	4.8

To show that the model can simulate conditions of different pathologies, a case study of aortic valve stenosis was simulated. The aortic valve area was decreased from 4.8 to 0.8 cm^2^ to represent non-existent and non-chronic severe aortic valve stenosis, respectively. Systemic flow and mean arterial pressure were maintained, whereas aortic valve stenosis-induced cardiovascular adaptations were not simulated to reduce model complexity. Simulated LAD flow velocity was compared to clinical measurements performed by Hildick-Smith and Shapiro (2000) before and 6 months after aortic valve replacement.

### Numerical Implementation

The set of differential equations describing pressure and volume has been solved numerically with a time step of 1 ms. Calculation time for simulation of one cardiac cycle in MATLAB 2019a (The MathWorks, Natrick, MA) on a standard personal computer with an Intel^®^ Core™ i7 processor and 16 GB RAM was approximately 2 s.

## Results

### Proximal Coronary Arterial Inflow and Distal Venous Outflow

Figure 3 shows the simulated flow velocity waves in the epicardial coronary arteries and veins, together with measured flow velocity waves (Davies et al., 2006; Hadjiloizou et al., 2006; Bloch et al., 2009; Moro et al., 2011). Visual agreement between model and measured waveforms was excellent in most cases.

The model predicted the typical biphasic flow velocity waveform in the left coronary arteries, with marked diastolic flow predominance (Figure 3A). In contrast, the flow waveform of the RCA was more uniformly distributed over the cardiac cycle, mainly due to lower intramyocardial pressure. Simulated peak diastolic-to-systolic flow velocity ratio as well as diastolic-to-total velocity time integral of the coronary arteries agreed well with reported average values (Table 3; Ofili et al., 1993, 1995; Akasaka et al., 1996; Crowley and Shapiro, 1997; Daimon et al., 2005; Hadjiloizou et al., 2006, 2008; Okura et al., 2006; Tani et al., 2009).

**TABLE 3:** Quantitative comparison of model arterial flow with *in vivo* measurements.

	Unit	Model	Measurement	
			Value	References
**Arterial peak diastole-to-systole velocity**				
RCA	–	1.2	1.0 ± 0.3	Hadjiloizou et al., 2008
			1.1 ± 0.2	Akasaka et al., 1996
			1.4 ± 0.2	Ofili et al., 1995
LM	–	1.3	1.8 ± 0.5	Hadjiloizou et al., 2008
LAD	–	2.3	2.1 ± 0.3	Tani et al., 2009
			2.3 ± 0.7	Okura et al., 2006
			2.0 ± 0.5	Daimon et al., 2005
			2.2 ± 0.8	Crowley and Shapiro, 1997
			2.2 ± 0.5	Ofili et al., 1995
LCx	–	2.1	1.8 ± 0.7	Ofili et al., 1995

**Arterial diastolic-to-total velocity time integral**				

RCA	–	0.7	0.6*	Hadjiloizou et al., 2008
			0.6*	Hadjiloizou et al., 2006
			0.6*	Ofili et al., 1995
LA	–	0.8	0.7*	Hadjiloizou et al., 2008
LAD	–	0.8	0.8	Crowley and Shapiro, 1997
			0.8*	Crowley and Shapiro, 1997
			0.8*	Ofili et al., 1995
LCx	–	0.8	0.7*	Ofili et al., 1995
			0.8*	Ofili et al., 1993

*Calculated from mean diastolic- and systolic time integral.

The coronary veins showed a systolic and diastolic phase of antegrade flow, separated by a short period of retrograde flow (Figure 3B), similar to measured coronary sinus flow data (Van Rossum et al., 1992; D’Cruz and Shirwany, 2003; Bloch et al., 2009). Average simulated distal coronary sinus flow of 143 mL/min was in range with reported values between 69 and 144 mL/min (Van Rossum et al., 1992; Schwitter et al., 2000; Koskenvuo et al., 2001; Watzinger et al., 2005; Kennedy et al., 2007; Bloch et al., 2009).

### Dynamics of Intramyocardial Flow and Diameter

**FIGURE 4:**
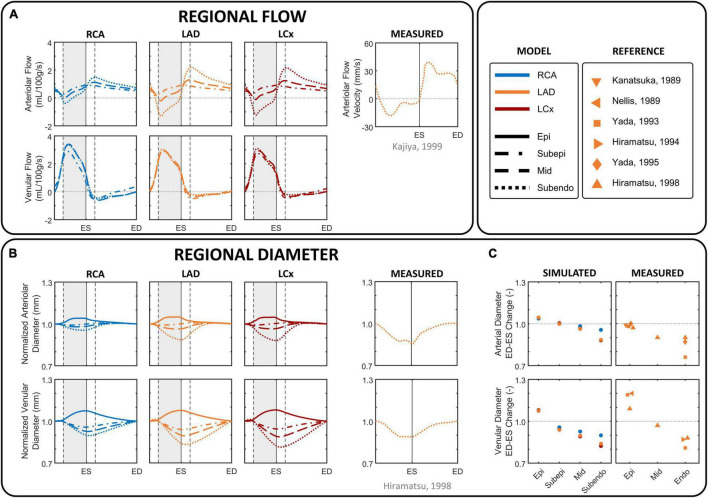
Phasic changes in regional microvascular flow and diameter of arterioles and venules in different coronary layers. **(A)** Simulated arteriolar (R1) and venular (R2) intramyocardial flow in the subepicardial, mid-, and subendocardial layer during one cardiac cycle, together with arteriolar subendocardial flow measured in the left anterior descending (LAD) (Kajiya and Goto, 1999). **(B)** imulated intramyocardial diameter in the epicardial, subepicardial, mid-, and subendocardial layer during one cardiac cycle, together with normalized subendocardial diameter measured in the LAD (Hiramatsu et al., 1998). Simulated diameter changes were derived from the arteriolar and venular volumes, assuming a circular shape and constant length, normalized by the diameter at end-diastole to facilitate the evaluation of the amplitude of diameter change over the cardiac cycle. **(C)** Simulated percental change in diameter of epicardial (Epi), subepicardial, mid, and subendocardial arterioles and venules from end-diastole (ED) to end-systole (ES), together with measured data in the LAD (Kanatsuka et al., 1989; Nellis and Whitesell, 1989; Yada et al., 1993, 1995; Hiramatsu et al., 1994, 1998). Vertical gray areas indicate left ventricular ejection and vertical dashed/solid lines indicate opening/closing of left ventricular cardiac valves. LCx, left circumflec artery; RCA, right coronary artery.

Simulated arteriolar flow exhibited some retrograde flow during early systole, slightly increasing velocities toward end-systole, and diastolic flow dominance (Figure 4A). In contrast, simulated venular flow was dominant in systole and almost absent throughout diastole. Because the magnitude of intramyocardial pressure varies transmurally, being greatest in the innermost myocardium and decreasing progressively across the ventricular wall, subendocardial arteriolar flow showed the most pulsatile behavior and exhibited a substantial degree of retrograde flow. Subepicardial arteriolar flow showed relatively similar magnitudes of antegrade flow during diastole and systole.

Similar to flow, arteriolar diameter varied most in the subendocardium (Figures 4B,C). The diameter of subendocardial arterioles in the right and left coronary branches decreased from end-diastole to end-systole with 4 and 12%, respectively. In contrast, the diameter of the subepicardial arterioles changed little during the cardiac cycle, whereas the diameter of the epicardial arterioles even increased 3–5% from end-diastole to end-systole.

Venular flow was systolic dominant, with similar magnitudes in all layers of a branch (Figure 4A). The diameter of subendocardial venules decreased 10–18% from end-diastole to end-systole, whereas the diameter of epicardial venules increased 7–8% from end-diastole to end-systole as blood is being squeezed out of the microcirculation (Figures 4B,C).

Model-predicted subendocardial LAD flow and diameter were in agreement with their corresponding phasic measurements (Kanatsuka et al., 1989; Nellis and Whitesell, 1989; Yada et al., 1993, 1995; Hiramatsu et al., 1994, 1998; Kajiya and Goto, 1999) as shown in Figure 4.

### Aortic Stenosis

**FIGURE 5:**
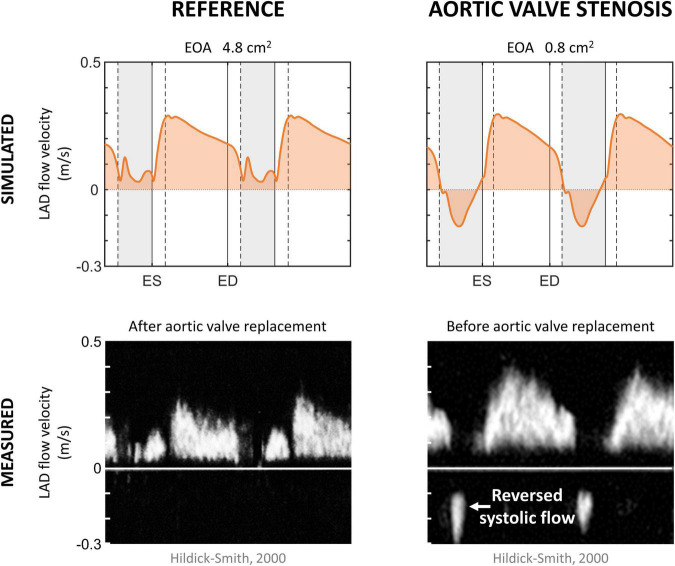
Aortic valve stenosis. Simulated left anterior descending (LAD) coronary artery flow velocity in the reference and aortic valve stenosis case, together with measured LAD flow velocity in patients 6 months after and before aortic valve replacement (Hildick-Smith and Shapiro, 2000). Vertical gray areas indicate left ventricular ejection and vertical dashed/solid lines indicate opening/closing of left ventricular cardiac valves. ED, end-diastole; EOA, effective orifice area; ES, end-systole.

Figure 5 shows the effect of simulated aortic valve stenosis on the predicted LAD flow velocity, together with transthoracic Doppler echocardiography measurements by Hildick-Smith and Shapiro (2000) before and after aortic valve replacement. A period of reverse systolic flow was present in both simulation and measurement with aortic valve stenosis. Simulated peak systolic flow velocity decreased from 0.13 m/s in the reference simulation to 0.04 m/s with aortic valve stenosis, consistent with the decreased peak systolic flow velocity of 0.16 ± 0.08 m/s after aortic valve replacement compared to 0.05 ± 0.11 m/s before aortic valve replacement found in the measurements (Hildick-Smith and Shapiro, 2000). Peak diastolic flow velocity remained unaffected in both the simulation and the measurements.

The aortic valve stenosis-induced increase in systolic intramyocardial pressure forced the coronary microcirculation to work at minimal diameters, reducing mean flow by 35% in the LAD (Table 4). As the intramyocardial pressure is greatest in the endocardium, the inner layers of the myocardium play a major role in the decreased mean systolic contribution to forward coronary flow, i.e., endo-to-epi ratio decreased.

**TABLE 4:** Comparison of reference model parameter values with the case of aortic valve stenosis.

	Unit	Reference	Aortic stenosis
**Input**			
Aortic valve orifice area	cm^2^	4.8	0.8

**Output**			

**General**			
Cardiac output	L/min	5.1	5.0
Mean arterial pressure	mmHg	91	91
Peak left ventricular pressure	mmHg	110	160
Mean pressure gradient	mmHg	1	45
Peak aortic flow velocity	m/s	0.9	4.0

**Mean coronary flow**			
Right coronary artery	mL/min	112	96
Left anterior descending	mL/min	83	54
Left circumflex	mL/min	60	40

**Endo-to-epi flow ratio**			
Right coronary artery	–	1.11	0.80
Left anterior descending	–	1.11	0.38
Left circumflex	–	1.11	0.39

## Discussion

The newly designed model of coronary mechanics combines a 1D coronary vascular network, lumped coronary microcirculation, and intramyocardial (pressure) components, to capture relevant details at each level of the coronary circulation. The coronary circulation was successfully implemented in the closed-loop CircAdapt model of the cardiovascular system, allowing simulation of coronary mechanics and hemodynamics in a wide variety of physiological and pathophysiological conditions. Model-predicted waveforms featured the main dynamic characteristics of epicardial and intramyocardial flow and vessel diameter of the coronary circulation. The versatility and validity of the model was demonstrated in a case study of aortic valve stenosis followed by valve replacement. The model independently reproduced the effects of aortic valve stenosis and valve replacement on coronary flow velocity measured in patients before and after valve replacement.

### Model Assumptions in Relation to Other Models

#### Overview Coronary Circulation

In the present study, a 1D wave-propagation model for the three main coronary branches was coupled to a 0D lumped parameter model of the small intramyocardial vessels, with extravascular compressive forces acting on the intramyocardial compliances. This modeling approach has been adopted by many others, although different degrees of complexity in coronary vascular network have been reported, ranging from tens to millions of 1D vessel segments and from a single to 31 0D transmural layers (Guiot et al., 1990; Rammos et al., 1998; Smith et al., 2002; Mittal et al., 2005; Huo and Kassab, 2007; Reymond et al., 2009; Mynard et al., 2010, 2014; Algranati et al., 2011; van der Horst et al., 2013; Mynard and Smolich, 2015, 2016; Ge et al., 2018, 2019; Duanmu et al., 2019; Scarsoglio et al., 2019; Fan et al., 2020, 2021). Though our model our model (with four arterial and five venous segments, and three transmural layers) does not describe the anatomical configuration in as much detail as recent models by Mynard and Smolich (2015, 2016) and Ge et al. (2018, 2019), the level of detail was based on data availability and the model could capture the main dynamic features of coronary hemodynamics at each level of the network. Due to the modular design of the CircAdapt model, the anatomical detail can be easily altered when needed. For example, modification of specific model parameter (values), e.g., the arbitrarily chosen vessel lengths, or inclusion of new input parameters, e.g., additional coronary vessel beds, is simple and would allow for e.g., investigation of the impact of a certain regional change.

#### One-Fiber Model for Myocardial Contraction

Different from previous mathematical models (Rammos et al., 1998; Reymond et al., 2009; Mynard et al., 2010, 2014; Algranati et al., 2011; Mynard and Smolich, 2015, Mynard and Smolich, 2016; Ge et al., 2018, 2019; Duanmu et al., 2019; Scarsoglio et al., 2019; Fan et al., 2020, 2021), where a time-varying elastance model was adopted to govern cardiac mechanics, our study adopted the one-fiber model of myocardial contraction (Arts et al., 1991). The main advantage of this particular model over time-varying elastance models is that it is based on geometric data (e.g., cavity and wall volume, wall thickness) in combination with microstructural properties of the myocardium (e.g., active and passive fiber stress development). Hence, with the one-fiber contraction model, global ventricular pump mechanics (volumes and pressures) are related to local myofiber mechanics (fiber stress and strain) (Lumens et al., 2009), allowing for easy simulation of cardiac diseases involving abnormal myocardial properties, ranging from hypertrophy to more complex pathologies like myocardial conduction disorders (Walmsley et al., 2015) or diastolic dysfunction (van Loon et al., 2020).

#### Myocardium-Vessel Interaction

*In vivo* measurement of the spatial and temporal distribution of the intramyocardial pressure is difficult because of uncontrolled distortion of the tissue microstructure by the pipette tip (Westerhof, 1990), the inhomogeneities of the pressure in various microcompartments (Spaan, 1995) and cardiac motion.

Several myocardium-vessel interaction mechanisms have been proposed to describe the intramyocardial pressure, but none by themself can account for all major coronary flow features. The first mechanism is CEP, which arises from transmission of ventricular cavity pressure into the myocardial wall. CEP is generally assumed to be equal to cavity pressure at the endocardium with an approximately linear decline to pericardial pressure at the epicardium (Westerhof et al., 2006; Spaan et al., 2008; Algranati et al., 2010; Mynard et al., 2014).

Subsequently, Krams et al. (1989) proposed the VE mechanism, emphasizing the importance of changes in ventricular elastance (i.e., stiffness), as CEP could not explain the similar coronary flow impediment in isovolumic and isobaric contractions (Downey et al., 1974; Carew and Covell, 1976; Marzilli et al., 1979). VE has been assumed proportional to ventricular elastance (Fan et al., 2020, 2021), or when using a one-fiber contraction model, to radial wall stress (Beyar et al., 1993; van der Horst et al., 2013). The advantage of the latter is that sarcomere properties are directly related to the intramyocardial pressure.

The third hypothesized mechanism was based on shortening-induced intracellular pressure (SIP), which arises from the thickening of shortening myocytes that leads to compression of adjacent blood vessels, as proposed by Rabbany et al. (1989, 1994). However, quantitative information on the effect of muscle shortening and thickening during contraction on coronary flow is lacking and the different approximations of SIP are debatable. Mynard and Smolich (2015, 2016) and Ge et al. (2018, 2019) both assumed SIP to be directly proportional to ventricular elastance, adopting a similar approach to the VE mechanism. Alternatively, SIP can be directly related to sarcomere stretch ratio (Algranati et al., 2010; Fan et al., 2020, 2021), defined as the ratio of cavity volume to end-diastolic volume. This approach, however, is questionable as the zero strain state changes with changes in end-diastolic volume.

The transmural difference in intramyocardial pressure cannot be explained by the SIP or VE mechanisms, whereas CEP alone cannot explain intramyocardial pressures being higher than cavity pressure, highlighting the need to combine CEP with VE and/or SIP. Due to the lack of data supporting the SIP mechanism, the abovementioned SIP issues, as well as the possibility to relate VE directly to sarcomere properties, the intramyocardial pressure in this study was assumed to be a summation of the CEP and VE mechanisms. The VE approximation adopted in this study is based on wall stress, taking into account contraction-dependent active stress, intrinsic elasticity, as well as passive stress generated by extracellular matrix and intracellular tissues like titin (Arts et al., 2005), as explained in more detail in Supplementary Material. This approach may give rise to more coronary flow impediment in early and mid-systole, compared to the approximation with SIP by ventricular elastance, which peaks in late-systole, although the effect is likely to be negligible in the healthy subject.

#### Coronary Territory Assignment

The standardized assignment of coronary territories to individual myocardial segments (Figure 1B) is currently based on empirical assumptions (Cerqueira et al., 2002). However, several studies have confirmed the oversimplicity of this mapping due to large anatomical variations of the coronary territories among individuals, especially in the apical region (Ortiz-Pérez et al., 2008; Termeer et al., 2010; Malkasian et al., 2020). The anatomical variation, in combination with the assumed flow distribution based on myocardial wall segment mass (Table 1), complicate quantitative comparison of model results with measurement data. Nevertheless, model-generated coronary flow waveforms matched well with measured waveforms in terms of mean flow, peak diastolic-to-systolic flow velocity ratio and diastolic-to-total velocity time integral (Table 3) given the standard coronary mapping. Modification of specific model parameters like wall (segment) mass and coronary mapping is simple and straightforward due to the modular design of the CircAdapt model and allows for simulation, e.g., of left and right coronary dominance.

#### Collapsible Tube Law

As the coronary vessels are subjected to high extravascular pressures, we applied a tube law (Equation 2) that, based on the work of Shapiro (1977) and Kozlovsky et al. (2014), takes into account vascular deformation at negative transmural pressures. Although circular vascular geometries were assumed, the collapsible tube law enabled the model to simulate the associated hemodynamic effects of intramyocardial vessels over a wide range of transmural pressures. The simulated amplitude of change in subendocardial nominal diameter over a cardiac cycle (12%) in the LAD agreed well with *in vivo* measurements (10–24%) (Kanatsuka et al., 1989; Nellis and Whitesell, 1989; Yada et al., 1993, 1995; Hiramatsu et al., 1994, 1998), to some extent proving the ability of our model to simulate deformation of intramyocardial vessels.

### Comparison of Model Simulations With Measurements

Available data that could be used for validation is scarce, especially for microvascular and venous sites. In addition, data from different sources are subject to considerable biological variability and may at times even contradict other studies. For these reasons, validation was carried out against qualitative waveforms measured in humans and a quantitative comparison was conducted only if sufficient *in vivo* data was available.

#### Reference Simulation

In general, the flow, velocity and diameter patterns predicted by the model were qualitatively in line with published *in vivo* data of healthy human controls. Specifically, the model performed well in predicting systolic and early diastolic flow transitions arising from wave propagation effects in the arterial tree (Figure 3A). Quantitatively, overall levels of peak systolic and peak diastolic velocity, caused by onset of ventricular ejection and relaxation, respectively, were similar in the model and experiments (Table 3).

Intramyocardial vessels are subjected to the phasic mechanical influences of cardiac contraction and relaxation, resulting in a unique blood flow pattern where arteriolar flow is diastolic predominant and venular flow is systolic predominant (Kajiya et al., 2000; Figure 4A). Sustained retrograde flow in subendocardial arterioles during systole was observed *in vivo* (Kajiya and Goto, 1999) as well as in our simulation. In some subjects, near zero flow was measured during the latter part of systole (Toyota et al., 2005). Subepicardial arterioles showed forward flow throughout the cardiac cycle, in agreement with *in vivo* measurements (Kajiya et al., 2000; Toyota et al., 2005), although the model did not predict early diastolic rise in subepicardial flow. A possible explanation for the small deviations in arteriolar flow patterns between model and experiments may be attributed to the simplicity of our coronary vascular network.

The coronary sinus typically shows a biphasic pattern (Figure 3B), although the physiological variation between subjects is large. Van Rossum et al. (1992) reported the systolic phase lower in amplitude compared to the diastolic phase, whereas the opposite was observed by D’Cruz and Shirwany (2003) and while both cases were reported by Bloch et al. (2009).

Despite the lack of intramyocardial venular flow data, the systolic venular flow dominance as predicted by the model (Figure 4A) is in agreement with measurements of myocardial venous outflow (Kajiya et al., 2000). The discrepancy between the *biphasic venous* flow pattern (Figure 3B) and the *monophasic venular* flow pattern (Figure 4A) can be explained by large venous compliance, corresponding low wave velocity, relatively high blood flow velocity and atrial contraction.

Simulated vascular diameter was qualitatively and quantitatively compared with *in vivo* LAD data (Nellis and Whitesell, 1989; Yada et al., 1993, 1995; Hiramatsu et al., 1994, 1998), confirming the model’s ability to predict phasic variations in vascular volume (Figures 4B,C).

#### Aortic Valve Stenosis

Simulated coronary flow wave patterns in aortic valve stenosis qualitatively agreed with the waveforms shown in literature (Hongo et al., 1993; Hildick-Smith and Shapiro, 2000), indicating that the model is able to capture the main phenomena contributing to altered coronary flow patterns in aortic valve stenosis while changing only a single parameter (i.e., valve area). More specifically, a period of reverse systolic flow was present as a result of the aortic valve stenosis-induced increase in systolic intramyocardial pressure. For an in-depth study, however, one should account for compensatory mechanisms that commonly occur when aortic valve stenosis progresses, such as LV hypertrophy and increased coronary flow demand (Hildick-Smith and Shapiro, 2000), as well as autoregulation. Inclusion of these mechanisms was beyond the scope of the current study.

### Clinical and Future Perspective

Our modeling framework enables simulation of key phenomena that contribute to coronary hemodynamics, such as cardiac, pericardial, valvular, systemic, pulmonary, vascular, and intramyocardial factors. In particular, the one-fiber model of myocardial contraction enables in-depth study of cardiac-to-coronary coupling, which is very difficult to achieve *in vivo*. For example, specific material properties can be changed per wall segment [e.g., asynchronous activation or local myocardial infarct (Lumens et al., 2008; Huntjens et al., 2014; Walmsley et al., 2015)] in order to study the effects of regional heterogeneity of myocardial tissue properties on coronary hemodynamics.

Besides application of this model as a research platform for *in silico* investigation of coronary hemodynamic changes under pathological conditions, it might serve as an educational tool to demonstrate the functional complexity of the coronary circulation.

The next step in improving the present model would be to include a bi-directional interaction of ventricular mechanics and coronary perfusion. This would be interesting when investigating large changes in perfusion pressure (Gregg, 1963) or impeded vascular emptying (Westerhof et al., 2006). Furthermore, inclusion of coronary autoregulatory mechanisms in future studies could be valuable to evaluate the effect of altered cardiac mechanics and myocardial demand on coronary flow reserve (Gould et al., 1990) under resting and hyperemic conditions. Our modeling framework with the one-fiber model for myocardial contraction provides an opportunity to match local coronary supply to local myocardial demand, the latter determined by myocardial stress and strain.

## Conclusion

We have presented a model of coronary mechanics that combines a relatively simple one-dimensional network of the major coronary vessels with a lumped microvascular model consisting of three transmural layers, to capture epicardial flow velocity as well as intramyocardial flow and diameter. Regional coronary perfusion was made dependent on myocardial contraction through the intramyocardial pressure, based on global pump mechanics and local myofiber mechanics. Coupled to the closed-loop CircAdapt model of heart and circulation, our modeling framework enables simulation of coronary hemodynamics. Qualitative and quantitative comparison of simulation results with clinical data showed that the model correctly described epicardial flow velocity, as well as intramyocardial flow and diameter. The versatility and validity of the model was demonstrated in a case study of aortic valve stenosis followed by valve replacement. The model independently reproduced the effects of aortic valve stenosis and valve replacement on coronary flow velocity measured in patients before and after valve replacement. In summary, we have presented a powerful multi-scale modeling framework that enables realistic simulation of coronary hemodynamics. This modeling framework can be used as a research platform for in-depth studies of cardiac-to-coronary coupling in healthy and diseased conditions.

## Data Availability Statement

The raw data and source code supporting the conclusions of this article will be made available by the authors, without undue reservation.

## Author Contributions

AM contributed to conception and design of the study, performed the simulations, and wrote the manuscript. All authors helped with analysis and interpretation of the data, contributed to manuscript revision, as well as read and approved the submitted version.

## Conflict of Interest

The authors declare that the research was conducted in the absence of any commercial or financial relationships that could be construed as a potential conflict of interest.

## Publisher’s Note

All claims expressed in this article are solely those of the authors and do not necessarily represent those of their affiliated organizations, or those of the publisher, the editors and the reviewers. Any product that may be evaluated in this article, or claim that may be made by its manufacturer, is not guaranteed or endorsed by the publisher.

## Footnotes

1
www.circadapt.org

## Abbreviations

CEP: cavity-induced extracellular pressure
IMP: intramyocardial pressure
LAD: left anterior descending coronary artery
LCx: left circumflex coronary artery
LM: left main coronary artery
LV: left ventricle
RCA: right coronary artery
SIP: shortening-induced intracellular pressure
VE: varying-elastance.
