# Supplementary material for: A Closed-Loop Modeling Framework for Cardiac-to-Coronary Coupling
Source: Front Physiol. 2022 Feb 28;13:830925. doi: 10.3389/fphys.2022.830925 (PMC8919076; doi:10.3389/fphys.2022.830925)
Supplement: Supplementary file 1 [file Data_Sheet_1.PDF]

Supplementary Material

1 Supplemental Methods

1.1 Model of myofiber mechanics

In the supplementary material, the relation between natural myofiber strain (ε_f) and Cauchy myofiber stress (σ_f) is presented.

Natural myofiber strain (ε_f) was converted to sarcomere length (L_s) by

$$L_s = L_{s,ref}\, e^{\varepsilon_f} \quad \text{(S1)}$$

with $L_{s,ref}$ the reference sarcomere length at zero myocardial strain. The sarcomere was represented by a passive elastic element in parallel with a series contractile and series elastic element. The time-dependent behavior was described by two state variables, i.e., contractile element length L_{sc} and mechanical activation C. This latter activation parameter is physiologically related to intracellular calcium concentration. The time derivative of L_{sc} depends linearly on length of the series elastic element ($L_s - L_{sc}$) and equals zero for isometric contraction:

$$\frac{dL_s}{dt} = v_{max}\left(\frac{L_s - L_{sc}}{L_{se,iso}} - 1\right) \quad \text{(S2)}$$

Where v_{max} represents velocity of sarcomere shortening with zero load and $L_{se,iso}$ length of the isometrically stressed series elastic element. Dependence on v_{max} represents the myofiber force-velocity relation so that shortening velocity increases with applied external force.

The time derivative of C is heuristically obtained, having separate terms to describe rise and decay of mechanical activation C:

$$\frac{dC}{dt} = \frac{1}{\tau_R} \cdot C_L(L_{sc}) \cdot F_{rise}(t) + \frac{1}{\tau_D} \cdot \frac{C_{rest} - C}{1 + e^{(T(L_{sc}) - t)/\tau_D}} \quad \text{(S3)}$$

Parameters τ_R and τ_D are scaling rise and decay time, respectively. Symbols t and C_{rest} represent time and diastolic resting level of activation, respectively. Functions C_L, F_{rise}, and T describe increase of activation with sarcomere length, rise of mechanical activation, and decrease of activation duration with decrease of sarcomere length, respectively:

$$C_L(L_{sc}) = \tanh(4.0(L_{sc} - L_{sc0})^2) \quad \text{(S4)}$$

$$F_{rise}(t) = 0.02 \cdot x^3 (8 - x)^2 e^{-x} \quad \text{(S5)}$$

with

$$x = \min\left(8, max\left(0, \frac{t}{\tau_R}\right)\right) \quad \text{(S6)}$$

$$T(L_{sc}) = \tau_{sc}(0.29 + 0.3L_{sc}) \quad \text{(S7)}$$

Where L_{sc0} and τ_{sc} represent contractile element length with zero load and a time factor scaling duration of contraction, respectively.

Active myofiber stress $\sigma_{f,act}$ depends on length of series elastic element, L_{sc}, and C:

$$\sigma_{f,act} = \sigma_{act} \cdot C \cdot (L_{sc} - L_{sc0}) \cdot \frac{L_s - L_{sc}}{L_{se,iso}} \quad \text{(S8)}$$

In order to simulate the contributions of titin as well as collagen to passive myocardial stiffness, passive stress $\sigma_{f,pas}$ represents a soft behaviour for low myofiber strain and a stiff behaviour for large positive strain:

$$\sigma_{f,pas} = \sigma_{pas}\left(36 \cdot \max\left(0, \varepsilon_f - 0.1\right)^2 + 0.1\left(\varepsilon_f - 0.1\right) + 0.0025e^{30\varepsilon_f}\right) \quad \text{(S9)}$$

The value of σ_{pas} has been adapted so that at maximum sarcomere length with exercise, passive myofiber stress assumes the maximum value. Total Cauchy myofiber stress σ_f is the sum of active and passive stress:

$$\sigma_f\left(\varepsilon_f\right) = \sigma_{f,pas} + \sigma_{f,act} \quad \text{(S10)}$$

1.2 Parameter values for the one-dimensional coronary network

The reference pressure (p_0) of the arterial and venous vessels were equal to mean arterial pressure and mean right atrial pressure, respectively, similar to systemic arterial and venous vessels. The reference lumen area (A_0) of the vessels was approximated based on their respective flow distribution. The determination of wall area (A_w) was set to be dependent on wall stress and impact velocity (i.e. velocity of waves generated when jumping) (Arts et al., 2005). The stiffness coefficient (k) was set to 11, similar to systemic proximal and distal vessels.

Supplementary Table 1 Parameter values for the one-dimensional network.

Vessel	Length Len (cm)	Reference lumen area A_0 (cm^2)	Wall area A_w (cm^2)
**Arterial vessels**			
RCA	2	0.109	0.039
LM	2	0.141	0.050
LAD	2	0.082	0.029
LCx	2	0.059	0.021
**Venous vessels**			
pCS	1	0.250	0.018
RCA	1	0.109	0.008
dCS	1	0.141	0.010
LAD	1	0.082	0.006
LCx	1	0.059	0.004

dCS: distal coronary sinus, LAD: left anterior descending, LCx: left circumflex, LM: left main, pCS: proximal coronary sinus, RCA: right coronary artery.

References

Arts, T., Delhaas, T., Bovendeerd, P., Verbeek, X., and Prinzen, F. W. (2005). Adaptation to mechanical load determines shape and properties of heart and circulation: The CircAdapt model. *Am J Physiol - Heart Circ Physiol* 288, 1943–1954. doi:10.1152/ajpheart.00444.2004.
